# Clinical Amyloid Typing by Proteomics: Performance Evaluation and Data Sharing between Two Centres

**DOI:** 10.3390/molecules26071913

**Published:** 2021-03-29

**Authors:** Diana Canetti, Francesca Brambilla, Nigel B. Rendell, Paola Nocerino, Janet A. Gilbertson, Dario Di Silvestre, Andrea Bergamaschi, Francesca Lavatelli, Giampaolo Merlini, Julian D. Gillmore, Vittorio Bellotti, Pierluigi Mauri, Graham W. Taylor

**Affiliations:** 1 Wolfson Drug Discovery Unit and National Amyloidosis Centre, Centre for Amyloidosis and Acute Phase Proteins, Division of Medicine, University College London, London WC1E6BT, UK; nigel.rendell@ucl.ac.uk (N.B.R.); p.nocerino@ucl.ac.uk (P.N.); j.gilbertson@ucl.ac.uk (J.A.G.); j.gillmore@ucl.ac.uk (J.D.G.); v.bellotti@ucl.ac.uk (V.B.); graham.taylor@ucl.ac.uk (G.W.T.); 2 Proteomics and Metabolomics Laboratory, CNR-ITB, Segrate, 20090 Milan, Italy; francesca.brambilla@itb.cnr.it (F.B.); dario.disilvestre@itb.cnr.it (D.D.S.); andrea.bergamaschi89@gmail.com (A.B.); pierluigi.mauri@itb.cnr.it (P.M.); 3 Amyloidosis Research and Treatment Centre, Fondazione IRCCS Policlinico San Matteo and University of Pavia, 27100 Pavia, Italy; francesca.lavatelli@unipv.it (F.L.); gmerlini@unipv.it (G.M.); 4 Department of Molecular Medicine, Institute of Biochemistry, University of Pavia, 10121 Pavia, Italy

**Keywords:** amyloid proteomics, proteomics platforms, proteomics results validation, LC-MS/MS raw data exchange

## Abstract

Amyloidosis is a relatively rare human disease caused by the deposition of abnormal protein fibres in the extracellular space of various tissues, impairing their normal function. Proteomic analysis of patients’ biopsies, developed by Dogan and colleagues at the Mayo Clinic, has become crucial for clinical diagnosis and for identifying the amyloid type. Currently, the proteomic approach is routinely used at National Amyloidosis Centre (NAC, London, UK) and Istituto di Tecnologie Biomediche-Consiglio Nazionale delle Ricerche (ITB-CNR, Milan, Italy). Both centres are members of the European Proteomics Amyloid Network (EPAN), which was established with the aim of sharing and discussing best practice in the application of amyloid proteomics. One of the EPAN’s activities was to evaluate the quality and the confidence of the results achieved using different software and algorithms for protein identification. In this paper, we report the comparison of proteomics results obtained by sharing NAC proteomics data with the ITB-CNR centre. Mass spectrometric raw data were analysed using different software platforms including Mascot, Scaffold, Proteome Discoverer, Sequest and bespoke algorithms developed for an accurate and immediate amyloid protein identification. Our study showed a high concordance of the obtained results, suggesting a good accuracy of the different bioinformatics tools used in the respective centres. In conclusion, inter-centre data exchange is a worthwhile approach for testing and validating the performance of software platforms and the accuracy of results, and is particularly important where the proteomics data contribute to a clinical diagnosis.

## 1. Introduction

The term “amyloidosis” is applied to a class of protein deposition diseases where misfolded proteins accumulate in form of insoluble fibrils in the extracellular space of several tissues. These deposits progressively lead to organ dysfunction, most frequently involving the heart, kidneys and central nervous system [1,2]. To date, more than 30 amyloidogenic proteins have been reported [3].

The clinical spectrum of amyloidosis is determined by the type of amyloidogenic protein and the affected organs. Early diagnosis and accurate amyloid typing are crucial since organ dysfunction increases with continuing amyloid deposition. An accurate diagnosis of amyloidosis involves the analysis of tissue biopsy from the affected organ or, alternatively, using the less invasive procedure of subcutaneous fat aspiration. Tissue biopsies are commonly formalin-fixed paraffin-embedded (FFPE), which is one of the most common methods for storing tissue samples. Collected samples are stained with Congo Red (CR) dye, and amyloid fibrils are detected by the typical birefringence under polarised light [4].

To identify the amyloid protein, immunological staining approaches, such as immunohistochemistry (IHC), have been proven to be the gold standard [5,6]. IHC has limitations in terms of specificity and sensitivity, depending on the type of amyloid and the available antibodies. However, despite its disadvantages, IHC is still the most common technique for identifying amyloid deposits.

More recently, in view of the IHC limitations, some clinical centres have started to rely entirely on mass spectrometry (MS)-based proteomics methods for amyloid typing [7,8,9]. MS-based proteomics has become a well-established approach [9,10,11] and is employed for both basic research and clinical diagnosis of amyloidosis. The use of laser capture microdissection (LCM) allows the precise selection of amyloid material (CR positive) for MS analysis, and LCM-MS is now considered a robust method for amyloid typing [9,11].

There are relatively few proteomics platforms dedicated to the analysis of amyloid around the world. The need to define common standard procedures and share experiences on several topics concerning amyloid proteomics and related methodologies led to the formation of the European Proteomics Amyloid Network (EPAN) in 2017. In this context, an inter-centre study focused on LC-MS/MS raw data exchange was carried out at National Amyloidosis Centre (NAC) in London and Istituto di Tecnologie Biomediche-Consiglio Nazionale delle Ricerche (ITB-CNR) based in Milan.

The NAC proteomics platform operates regularly as a clinical diagnostic test for amyloidosis and also for research into the pathogenesis of the disease. Since 2012, more than 2000 clinical samples, which include various tissue types, have been analysed by MS. The experience of the NAC in running a UK-accredited amyloid proteomics service to type amyloid, together with the benefits and limitations of the approach, have recently been reported [11]. Proteomics results are directly linked to the patient database, and by means of an algorithm it is possible to automatically identify the most likely amyloidogenic protein [11]. The common amyloidogenic proteins identified by proteomics in NAC’s patient database are apolipoproteins ApoA-I, ApoA-IV, ApoC-II, ApoC-III, atrial natriuretic peptide, fibrinogen Aα chain (FibAα), gelsolin (GSN), immunoglobulin light chains κ and λ, heavy chain, insulin, leukocyte cell-derived chemotaxin-2 (LECT2), lysozyme (LYZ), β2-microglobulin (B2M), semenogelin, serum amyloid A (AA) and transthyretin (TTR). The amyloidogenic status of galectin-7 has recently been challenged [12], and this is now under consideration by our consortium. NAC proteomics facility has recently been formally accredited by the UK Accreditation Scheme (UKAS) as part of the National Amyloidosis Centre’s diagnostic services [11].

ITB-CNR has applied gel-free proteomics to study amyloidosis since 2008 in collaboration with Hospital San Matteo (HSM) in Pavia. In particular, it has mainly analysed fat aspirate samples, and liver and cardiac tissues, supplied by HSM. Of note, analysed samples concern critical cases unsolved by IHC and are prepared without LCM. ITB-CNR developed the α-value algorithm to diagnose the four main types of amyloidosis, AL lambda and kappa, and TTR and AA, based on label-free approach [10]. Additionally, ITB-CNR applies systems biology approach to connect the thousands identified proteins into functional networks [13].

The present work is focused on the comparison of the amyloid proteomics results obtained in the two centres based in London and Milan. We report our experience of exchanging the mass spectrometry raw data for evaluating the quality and the confidence of our results achieved through the use of different software platforms and algorithms for amyloid protein identification.

## 2. Results

In the context of EPAN data exchange working group, forty LC-MS/MS raw data files were sent from NAC to ITB-CNR in order to be re-processed with their bioinformatics tools.

Mass spectrometer raw data of seven fat aspirates and thirty-three FFPE samples from different tissue types (Table 1) were selected from NAC database for re-analysis by ITB-CNR proteomics platform. The clinical information on the patients was not provided in order to blind the analysis. These had been previously typed by proteomics together with biochemical (morphology, IHC and genetics) and clinical presentation as AL (κ) (5), AL (λ) (10), ApoA-I (1), ApoA-IV (1), B2M (1), DNJB9, a marker of fibrillary glomerular nephritis (FGN) (2), fibrinogen Aα (2), heavy chain (2), insulin (1), LECT2 (1), lysozyme (2), SAA (2), semenogelin (1) and TTR (6). Samples were also included where the identification was uncertain (1) and where no amyloid signatures were detected (2). Raw data were re-analysed by Proteome Discoverer software and updated α-value algorithm; specifically, the four main amyloidosis proteins were extended to include seven others (LECT2, lysozyme, semenogelin, heavy chain, insulin, DNJB9 and fibrinogen).

**Figure 1:**
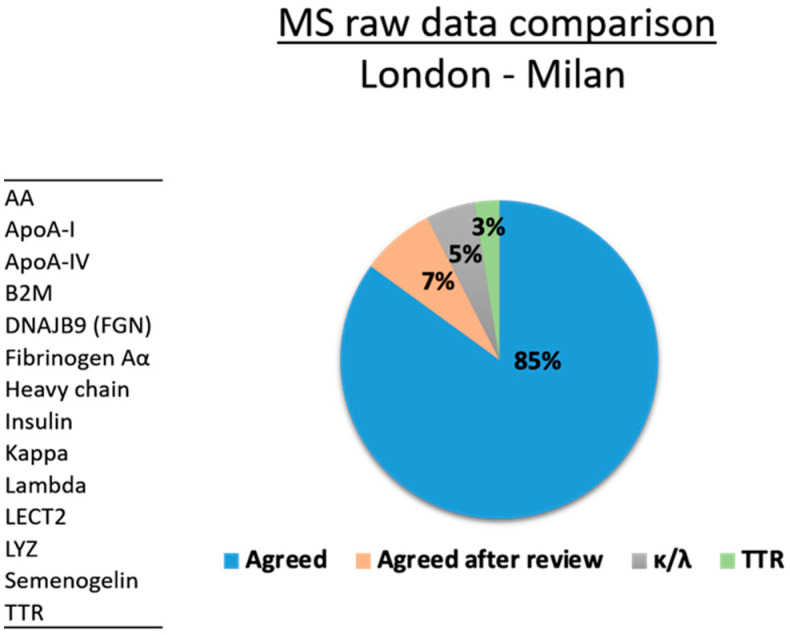
Mass Spectrometry (MS) raw data comparison between London (NAC) and Milan (ITB-CNR) proteomics platforms.

The results are shown in Table 1. Proteomics data agreed for 34/40 samples (85%) (Figure 1). These included AL (κ) (3), AL(λ) (10), ApoA-IV (1), B2M (1), DNJB9 (2), fibrinogen Aα (2), heavy chain (2), insulin (1), LECT2 (1), lysozyme (2), SAA (2), semenogelin (1) and TTR (6) cases (Table 1). In three cases there was an apparent conflict because the ITB-CNR definitions did not originally include “no amyloid signature” or “uncertain”, and samples were initially typed on the most obvious protein identification. On re-examination, there was agreement in three further samples: in the Congo red negative sample (#35, #40, Table 1) neither laboratory detected the amyloid signatures (SAP, ApoA-IV and ApoE) although other proteins (FibAα, kappa and lambda) were present, and in the one uncertain sample (#3, Table 1) multiple amyloid proteins were identified in both laboratories (Supplementary Tables S1 and S2).

There were 3/40 cases where the ITB-CNR and NAC results were not in agreement. Two NAC AL (κ) cases (#21 and #33, Table 1) were reported as ambiguous AL (κ/λ) by the ITB-CNR algorithm since both immunoglobulin κ and λ were detected with a very similar α-values. An ApoA-I sample (#25, Table 1) was classified as TTR by ITB-CNR proteomics group, although ApoA-I was identified with higher α-value (207) than TTR (72). Mascot analysis at NAC identified ApoA-I as the top scoring protein (3877). Other potential amyloidogenic proteins, such as TTR and immunoglobulin κ, were detected at NAC with much Mascot lower scores (320 and 113, respectively). The clinical and biochemical data were also consistent with ApoA-I amyloidosis.

## 3. Discussion

Sharing experimental procedures and discussing best practices is a remarkable approach to evaluate and improve methods, the performance of software platforms and the accuracy of amyloid proteomics analysis from the sample preparation to the mass spectrometry data processing. The collaboration between amyloid proteomics platforms can help to standardise procedures and narrow knowledge gaps on the natural history of the disease.

Here, we report an inter-centre validation study comparing proteomics data obtained through different software platforms and bioinformatics tools.

Our work showed a high concordance (92.5%, 37/40 total samples) between the proteomics data obtained in London and Milan, part of European Proteomics Amyloid Network (EPAN). This study demonstrated an excellent level of performance of the different bioinformatics tools used by London and Milan proteomics centres.

In a small proportion of NAC MS raw data analysed at ITB-CNR, the results disagreed. In some cases, this arose from a difference in reporting procedures. At the NAC, we report samples as no amyloid signature in cases where only one of the Mayo Clinic’s signature proteins (SAP, ApoA-IV and ApoE) is present. We currently do not include vitronectin as a signature protein even though it has been proposed as a signature protein [14,15] and is present in the majority of NAC amyloid samples. Similarly, where more than one amyloid protein is present with similar scores, and in the absence of other clinical or biochemical markers, we would determine the sample as uncertain rather than select the highest scoring protein. With AL patients, both immunoglobulin κ and λ are often present, and the NAC diagnosis of AL (κ) or AL (λ) is based on the light chain constant region score, moderated by the inclusion of any variable light chain regions that are present, together with clinical factors such as the presence of a light chain clone. Not all of this information was available at ITB-CNR. These data seem to indicate that the α-value alone is insufficient to distinguish between AL (κ) and AL (λ) amyloid. However, the α-value requires the evaluation of AL kappa and lambda levels in the specific reference tissues for determining the baseline noise. In particular, the two ambiguous cases were related to lung parenchyma and skin samples, never analysed for characterizing the baseline noise. Identifying ApoA-I and heavy chain amyloid purely by proteomics can be challenging since they can both be found in many Congo-red-positive tissue samples. ApoA-I is not usually included in the ITB-CNR α-value algorithm for amyloid classification and, in the absence of clinical and other data, it was misidentified as the next highest scoring protein, TTR. Since TTR amyloid is common in the elderly, the identification of TTR as a co-deposited amyloid protein cannot be excluded.

Of note, when α-value was updated with additional amyloid proteins, such as lysozyme, insulin and semenogelin, the identification of amyloidosis subtyping resulted in agreement with NAC findings.

Although the two centres applied different procedures in terms of search engine platforms and algorithms, the comparison allowed a very good concordance (>92%).

These findings indicate that the MS-based approach is robust, sensitive and less affected by biases than antibody-based methods. The availability of untargeted proteomic profiles permits the re-evaluation of data and the consideration of new subtypes. This is useful for the definition of different panels composed of different biomarkers leading to a high-precision diagnosis and the eligibility of the patients to specific therapeutic treatments, translating basic research to real-life and transforming medicine from evidence-based to personalised.

This is the first inter-laboratory comparison of amyloid proteomics raw data analysed using different search engines, different analysts and applying the algorithms currently in use at each centre. This approach, which was initiated at the first European Proteomics Amyloid Network meeting in London in 2017, offers a simple and inexpensive model for future accreditation studies.

## 4. Materials and Methods

**Figure 2:**
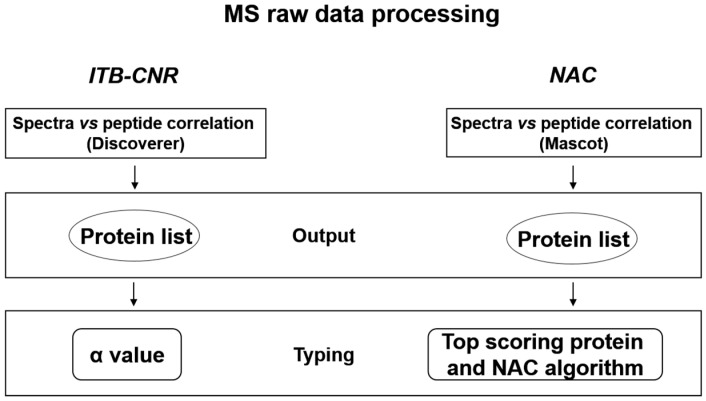
Scheme of proteomics data analysis at ITB-CNR and NAC facilities.

A scheme of NAC and ITB-CNR proteomics data analysis workflow is shown in Figure 2.

### 4.1. Proteomics Analysis at NAC

FFPE tissue biopsies and unfixed fat aspirates were obtained from patients attending the UK NHS National Amyloidosis Centre and also received from other clinical centres for immunochemical and proteomics characterization. Proteomics analysis procedure has been previously described in detail [11]. MS raw data were processed by Mascot software (Matrix Science, London, UK) using the Swiss-Prot human database, together with additional amyloid protein variant databases. Mascot search parameters were trypsin as proteolytic enzyme; two missed cleavage sites; MS tolerance of 10 ppm; 0.6 Da for MS/MS fragments; methionine oxidation as variable modification; N-methyl lysine included as variable modification when required [16]; included charge states +2, +3 and +4; and a significance threshold at *p* < 0.05. Proteomics results are linked to the NAC database, and the most likely amyloidogenic protein is displayed by using an algorithm, which has been previously described [11].

In addition, Mascot output data were also analysed and validated by running Scaffold 4.9.0 (Proteome Software, Inc., Portland, OR). Scaffold filtering parameters for protein identification were protein threshold confidence level >99%, with a minimum of two assigned peptides and a probability >95%.

LC-MS/MS raw data of thirty-three FFPE and seven fat aspirates were selected from NAC database in order to be re-analysed by ITB-CNR centre.

### 4.2. Proteomics Data Analysis at ITB-CNR

MS raw data obtained by NAC were processed by Discoverer 1.4 software, based on SEQUEST algorithm. Matches between spectra were only retained if they had a minimum Xcorr of 2.0 for +1, 2.5 for +2 and 3.5 for +3 charge state, respectively; protein rank was fixed to 1, while peptide confidence was fixed to “high”. In addition, the FDR was set to <5%. For amyloidosis subtyping, which involves evaluating which specific amyloid protein was prevalent in each patient, a parameter (α-value) was calculated; this was obtained by normalizing the patient over control ratio (>3) of each biomarker’s spectral count [10]; α-value was updated with amyloid proteins suggested by NAC deducing baseline value for each amyloidogenic protein from non-specific-subtype affected samples.

## Figures and Tables

**Table 1:** National Amyloidosis Centre (NAC) proteomics data analysed by Istituto di Tecnologie Biomediche-Consiglio Nazionale delle Ricerche (ITB-CNR). The Table shows the proteomics results obtained by the single centres, and the analysed tissue types: formalin-fixed paraffin-embedded (FFPE) biopsies (samples from 1 to 33), fat aspirates (samples from 34 to 40), and the agreement of final results between the two centres.

Sample No.	NAC	ITB-CNR	Tissue Type	Agreed
1	LECT2	LECT2	renal	Y
2	LYZ	LYZ	renal	Y
3	Uncertain	LYZ/AL (λ)/Heavy chain	renal	Y
4	AL (λ)	AL (λ)	renal	Y
5	AA	AA	renal	Y
6	DNJB9 (FGN)	DNJB9 (FGN)	renal	Y
7	DNJB9 (FGN)	DNJB9 (FGN)	renal	Y
8	Fibrinogen Aα	Fibrinogen Aα	renal	Y
9	Heavy chain	Heavy chain	renal	Y
10	AL (κ)	AL (κ)	renal	Y
11	Fibrinogen Aα	Fibrinogen Aα	renal	Y
12	AL (λ)	AL (λ)	cardiac	Y
13	TTR	TTR	cardiac	Y
14	TTR	TTR	cardiac	Y
15	ApoA-IV	ApoA-IV	cardiac	Y
16	TTR	TTR	myocardial	Y
17	Insulin	Insulin	soft tissue	Y
18	AL (λ)	AL (λ)	soft tissue	Y
19	Heavy chain	Heavy chain	soft tissue	Y
20	AL (λ)	AL (λ)	lung parenchyma	Y
21	AL (κ)	AL (κ)/(λ)	lung parenchyma	N
22	B2M	B2M	liver	Y
23	LYZ	LYZ	liver	Y
24	AL (λ)	AL (λ)	salivary gland	Y
25	ApoA-I	TTR	bone marrow trephine	N
26	AA	AA	thyroid	Y
27	AL (λ)	AL (λ)	jejunal	Y
28	TTR	TTR	pancreas	Y
29	Semenogelin	Semenogelin	prostate	Y
30	AL (κ)	AL (κ)	vocal cord	Y
31	AL (κ)	AL (κ)	tongue	Y
32	AL (λ)	AL (λ)	urethral	Y
33	AL (κ)	AL (κ)/(λ)	skin	N
34	AL (λ)	AL (λ)	fat aspirates	Y
35	No Amyloid Signature	FibAα/AL (κ)/no Amyloid Signature	fat aspirates	Y
36	AL (λ)	AL (λ)	fat aspirates	Y
37	AL (λ)	AL (λ)	fat aspirates	Y
38	TTR	TTR	fat aspirates	Y
39	TTR	TTR	fat aspirates	Y
40	No Amyloid Signature	AL(λ)/FibAα/DNAJB9/no Amyloid Signature	fat aspirates	Y

## Data Availability

Anonymised raw mass spectra data are available on request from the corresponding author.

## Supplementary Materials

The following are available online, Table S1: NAC raw data analysed at ITB-CNR, Table S2: NAC raw data analysed at NAC.

## Sample Availability

Clinical biopsies and digests are not available.
